# Treeline dynamics in response to climate change in the Min Mountains, southwestern China

**DOI:** 10.1186/1999-3110-54-15

**Published:** 2013-08-22

**Authors:** Zhi-Jiang Zhao, Guo-Zhen Shen, Liu-Yi Tan, Dong-Wei Kang, Meng-Jun Wang, Wen Kang, Wen-Xia Guo, Melanie JB Zeppel, Qiang Yu, Jun-Qing Li

**Affiliations:** 1 Key Laboratory for Silviculture and Conservation of MOE, School of Forest Science, Beijing Forestry University, Beijing, 100083 China; 2 State Key Laboratory of Vegetation and Environmental Change, Institute of Botany, Chinese Academy of Sciences, Beijing, 100093 China; 3 Department of Biological Sciences, Macquarie University, Sydney, NSW 2109 Australia; 4 Plant Functional Biology & Climate Change Cluster, School of the Environment, University of Technology, Sydney, NSW 2007 Australia

**Keywords:** *Abies faxoniana*, Age structure, Climatic variability, Radial growth, Treeline, Tree-ring chronology

## Abstract

**Background:**

*Abies faxoniana* is the dominant plant species of the forest ecosystem on the eastern edge of Qinghai-Tibet Plateau, where the treeline is strongly defined by climate. The tree-ring chronologies and age structure of *Abies faxoniana* were developed in the treeline ecotones on the northwestern and southeastern aspects of the Min Mountains in the Wanglang Nature Reserve to examine the treeline dynamics of recent decades in response to climate change.

**Results:**

On the northwestern aspect, correlation analysis showed that the radial growth was significantly and positively correlated with precipitation in current January and monthly mean temperature in current April, but significantly and negatively correlated with monthly mean temperature in previous August. On the southeastern aspect, the radial growth was significantly negatively correlated with monthly mean temperature in previous July and August.

**Conclusions:**

The different responses of radial growth to climatic variability on both the aspects might be mainly due to the micro-environmental conditions. The recruitment benefited from the warm temperature in current April, July and September on the northwestern aspect. The responses of radial growth and recruitment to climatic variability were similar on the northwestern slope. Recruitment was greatly restricted by competition with dense bamboos on the southeastern aspect.

**Electronic supplementary material:**

The online version of this article (doi:10.1186/1999-3110-54-15) contains supplementary material, which is available to authorized users.

## Background

Upper treeline ecotones within alpine and arctic ecosystems are mainly controlled by climate (e.g. Lavoie and Payette, 1994; Kullman, 1999; Grace et al., 2002), and are considered as sensitive proxy biomonitors for revealing the impact of climate variability on the distribution of high-elevation mountain forests (e.g. Camarero and Gutiérrez, 2004; Danby and Hik, 2007; Elliott, 2011). Many studies have shown that climatic variables limit the tree’s radial growth and recruitment at altitudinal treelines (Cullen et al., 2001; Takahashi et al., 2003; Wilmking et al., 2004; Elliott and Kipfmueller, 2011). The close relationships between climatic factors and tree radial growth are widely used to reconstruct past patterns of climate changes (e.g. Fritts, 1976; Cullen et al., 2001; Liu et al., 2009). The frequency of seedling recruitment at treeline ecotones can also reflect climatic change well (e.g. Kullman, 1993; Daniels and Veblen, 2004).

Various studies have revealed that treeline position is sensitive to temperature changes and climate warming has caused an increase in treeline elevation over time (e.g. Brubaker, 1986; Lloyd and Fastie, 2003; Danby and Hik, 2007; Leonelli et al., 2011) and is likely to cause further increases in treeline in the future (Munier et al., 2010). Increasing temperatures can provide a possible mechanism for abrupt increases in recruitment (Hessl and Baker, 1997; Elliott and Kipfmueller, 2011). Tree growth and survival at some upper timberlines are strongly limited by the low-temperature among the main factors controlling the treeline altitude (Tranquillini, 1979; Stevens and Fox, 1991; Körner, 2003; Holtmeier, 2009). In some altitudinal regions, radial growth of trees is driven by summer temperatures (LaMarche and Fritts, 1971; Eckstein and Aniol, 1981; Schweingruber et al., 1991; Bradley and Jones, 1993). There have been strong links between increased recruitment and warmer temperatures during the growing season and the cool seasons at treeline ecotones (Elliott and Baker, 2004; Danby and Hik, 2007; Holtmeier and Broll, 2007; Harsch et al., 2009; Kullman and Öberg, 2009).

As well as temperature, precipitation can also influence the treeline dynamics greatly. Global warming could exacerbate possible water limitations (Andersen et al., 2009) and cause heat-induced moisture stress without a concomitant increase in precipitation at altitudinal treeline (Weisberg and Baker, 1995; Daniels and Veblen, 2004). It has shown that moisture stress could limit seedling recruitment (Hessl and Baker, 1997; Lloyd and Graumlich, 1997) and tree growth (Jacoby and D’Arrigo, 1995; Barber et al., 2000; Lloyd and Fastie, 2002) at some upper treelines. Trees may lose the ability to grow continuously in the warmer temperature condition if insufficient soil water leads to drought stress, therefore precipitation may show a high positive correlation with tree ring-width (Bunn et al., 2005). Inversely, less precipitation in late spring and early summer may favor tree establishment by prolonging the growing season (Elliott and Kipfmueller, 2011).

It has been suggested that dendroclimatic data alone cannot determine the causes of changes in the structure of ecosystems and populations (Moiseev, 2002). In fact, tree radial growth and seedling recruitment are interrelated and must be considered together in order to gain an accurate understanding of treeline dynamics. Studies have uncovered treeline dynamics in relation to climatic change at the population and community level by studying dendroecological techniques coupled with stand age structures, climatic factors and ecological attributes (Ruffner and Abrams, 1998; Daniels and Veblen, 2004; Bunn et al., 2005; Wang et al., 2006; Jump et al., 2007; Elliott and Kipfmueller, 2011). At some altitudinal treelines, the climate conditions that facilitate the radial growth are similar to those that are conductive to recruitment (e.g. Szeicz and Macdonald, 1995; Camarero and Gutiérrez 1999; Gervais and MacDonald, 2000; Jump et al., 2007; Dang et al., 2009). Yet, the two processes of recruitment and growth may respond differently to climatic factors in some other treelines (e.g. Earle, 1993; Daniels and Veblen, 2004; Wang et al., 2006). The sensitivity of treelines to climate change varies with local and regional topographical conditions and thus differs as to its extent, intensity and the process of change (Holtmeier and Broll, 2005).

In mountainous areas, slope aspect has been considered as an important role for exploring the variability of upper treelines to climate change (e.g. Danby and Hik, 2007; Dang et al., 2009; Elliott and Kipfmueller, 2010; Elliott and Kipfmueller, 2011). For instance, soil moisture conditions on different slopes may exert notable differences in the spatiotemporal patterns of tree regeneration at upper treelines (e.g. Daniels and Veblen, 2004; Elliott and Kipfmueller, 2011). Treeline elevation and stand density may increase differently between slope aspects due to the differential presence of permafrost (e.g. Danby and Hik, 2007). The environmental factors mediated by slope aspect should be considered when assessing possible treeline response to climate change (Elliott and Kipfmueller, 2010).

To date, the manner by which climate variability affects radial growth and seedling recruitment of many upper treeline species in different geographic locations is not completely understood (Wang et al., 2006; Dang et al., 2009). Qinghai-Tibet Plateau (QTP) is considered as one of the most sensitive areas to global climate change in China (Hou et al., 2008). Some dendrochronological studies have been conducted in the northeastern QTP (Liu et al., 2006; Li et al., 2008; Fang et al., 2009), but few of these have explored the main climatic factors of temperature and precipitation to determine how each (alone and in combination) influence the subalpine treeline on the eastern edge of QTP. Based on recent data, the temperature in QTP has increased significantly over the past 50 years (Ding et al., 2009). Thus, the study presented herein was designed to determine how radial growth and recruitment of *Abies faxoniana* responded to the variability of temperature and precipitation on both northwestern and southeastern aspects in the Min Mountains on the eastern edge of QTP. We predicted that: climate warming enhanced both the radial growth and the seedling recruitment of *A. faxoniana* in the latest decades.

## Methods

### Study area

**Figure 1:**
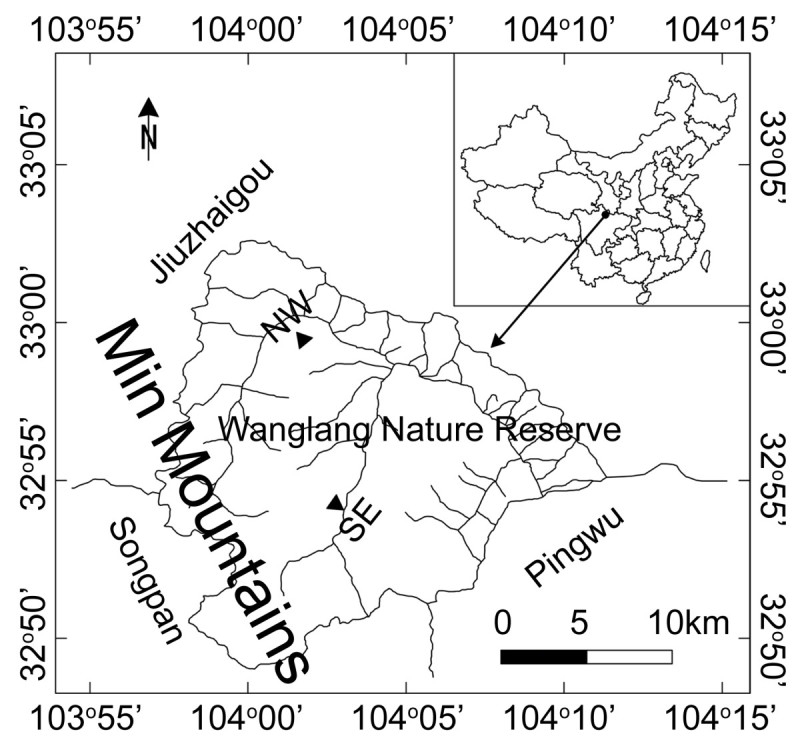
**Locations of sampling sites (▲) at the treeline ecotones on the northwestern (NW) and southeastern (SE) of the Min Mountains in the Wanglang Nature Reserve, southwestern China.**

This study was conducted in the Wanglang National Nature Reserve (32°49′-33°02′N, 103°55′-104°10′E) in the Min Mountains, on the eastern edge of QTP in western Sichuan Province, southwestern China (Figure 1). Elevations in the reserve range from 2300 to 4980 m. The terrain is steep and deeply dissected, with sharp environmental gradients.

**Figure 2:**
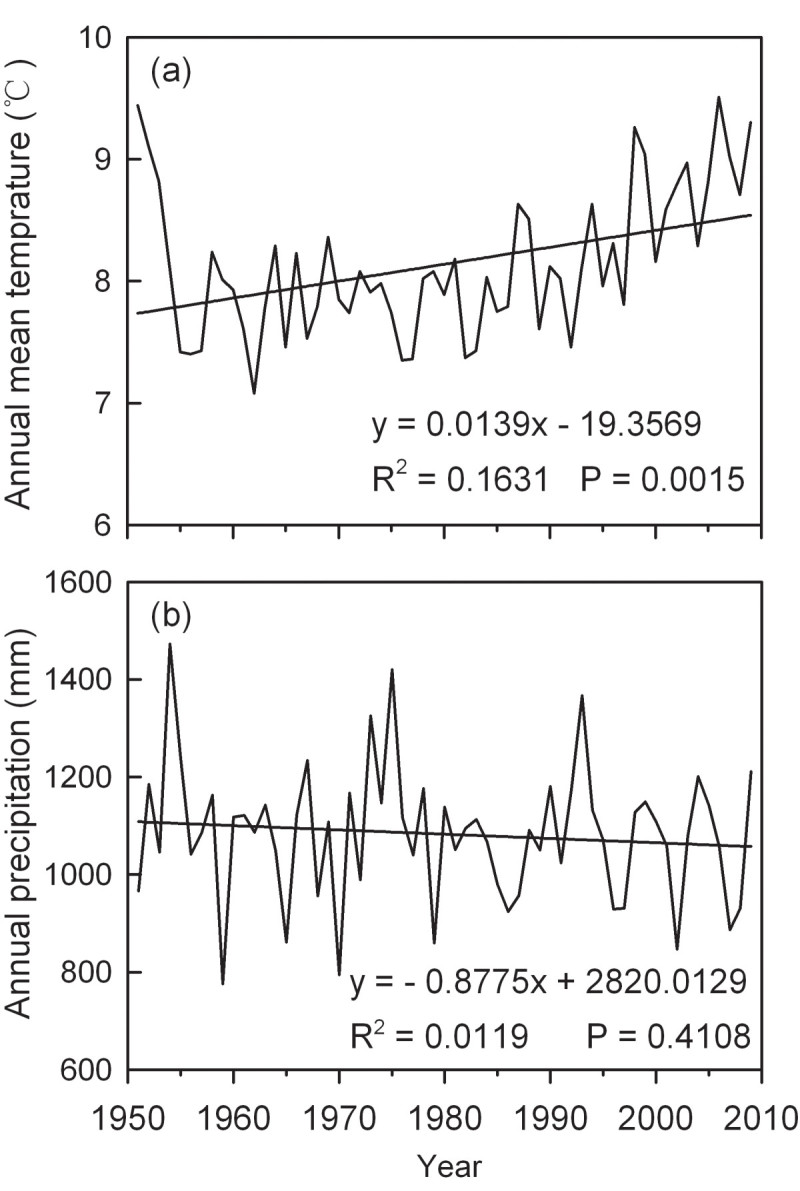
**Variations in annual mean temperature (a), annual precipitation (b) during the period 1951–2009 at the study area, calculated with data from Songpan meteorological station and using MTCLIM program.**

The Wanglang National Nature Reserve belongs to Danba-Songpan semi-humid climate, which is characterized by dry, cold winters and wet, cool summers. The mean annual temperature is 2.3°C and the mean annual total precipitation is about 1100 mm (Taylor et al., 2006). The average July temperature is 12.7°C and the average January temperature is −6.1°C, with a recorded extreme high temperature of 26.2°C and extreme low temperature of −17.8°C. The annual rainfall time may last more than 195 days and is concentrated in May, June and July. Between 1951 and 2009, the annual mean temperature increased at an average rate of 0.0137°C /yr (*R*^*2*^ = 0.1631, *P* = 0.0015) (Figure 2a). However, insignificant descending trend for the annual precipitation was found during this period (Figure 2b).

The subalpine fir, *A. faxoniana*, is the dominant coniferous tree species in the study area, having the most widely distributed range and largest volume. Most of the reserve from an elevation of ~2700 m to the upper treeline is covered by this species. The conifer forests below 2700 m were clear-felled in the 1950s, and some separate trees on or near the valley bottoms above this altitude were also cut until the reserve was established in 1964 (Taylor et al., 2006). However, the uppermost trees, often situated in fairly inaccessible locales, have remained generally undisturbed by humans or livestock. The treeline forests have been generating without disturbance, with no evidences of fires or previous logging.

### Site selection and field sampling

From June to August of 2010, two plots (each was 100 m × 20 m, 0.2 ha) were established at the subalpine treeline ecotones on the northwestern (32°59′27.3″ N, 104°01′44.0″ E, 3297 m a.s.l.) and southeastern (32°54′09.5″ N, 104°02′51.6″ E, 3,225 m a.s.l.) aspects of the Min Mountains in the Wanglang Nature Reserve (Figure 1). The main reason for choosing contrasting slope aspects is that the main mountain ridges all run northeast to southwest in this area. Both plots on northwestern and southeastern aspects can represent the treeline ecotones exactly here. The longer side of each plot was parallel to the isoline. The plots were selected based on the criterion that they should represent the fir forest structure at subalpine treeline.

In the plot, *A. faxoniana* individuals were divided into three height classes (Wang et al., 2006): trees (> 2 m), saplings (0.5-2 m) and seedlings (< 0.5 m). The d.b.h. (diameter at breast height) of each fir tree in the plot was measured. At least one core of each tree was extracted at breast height (1.3 m above the ground) in the direction parallel to the contour line, using an increment borer. One additional core was extracted from the opposite side of some trees. These trees included the ones which were selected for developing chronologies and the ones with broken or rotten increment cores. For all the saplings and seedlings in the plot, the number of branch whorls and bud scars on the main stem was counted and recorded (Daniels and Veblen, 2004). In total, 208 increment cores from 119 trees were sampled and 615 saplings and seedlings were measured at both the subalpine treelines. In addition, 68 saplings and seedlings with normal growth were randomly uprooted after recording their height and the number of whorls and scars, and cross-sectional disks were cut from their base stems to determine their accurate ages. The time required for seedlings to reach the d.b.h. was estimated through the age-height regression (Dang et al., 2009). Shade-tolerant species can vary greatly in growth rate, for example, individuals in the seedling bank of some *Abies* species typically grow slowly and can persist for a very long period (Antos et al., 2005). Consequently, fir seedlings and saplings with severe release or suppression events (Nowacki and Abrams, 1997) were excluded from developing age-height regression (Dang et al., 2010).

### Climate data

Climate data (1951–2009) were obtained from the nearest meteorological station, Songpan (32°39′N, 103°34′E, 2,580 m a.s.l.), approximately 55 km southwest of the sampling sites. Monthly mean temperature and monthly total precipitation of the sampling sites were simulated by Mountain Climate Simulator (MTCLIM, version 4.3; School of Forestry, University of Montana, Bozeman) based on daily maximum and minimum temperature and precipitation of base station (Thornton et al., 1997; Dang et al., 2009).

### Chronology development

In the laboratory, all the increment cores were mounted in slotted wooden boards, air-dried and sanded with successively finer sandpaper to produce a polished transverse surface for visual cross-dating (Stokes and Smiley, 1996). The annual ring-widths were measured to the nearest 0.01 mm, and the rings were counted using a LINTAB II measuring system. False rings, missing rings or measurement mistakes were all identified by cross-dating the patterns of wide and narrow rings among trees with the software package TSAP-Win (Rinn, 2003). The quality of cross-dating was controlled using the software COFECHA (Holmes, 1983). Cores which were too short, fragmented or rotted were discarded in order to improve the common signals in tree ring-width sequences. In total, 73 cores from 38 trees and 59 cores from 41 trees were used to build the chronologies for the northwestern and southeastern treelines, respectively.

The ring-width chronologies were standardized using the program ARSTAN (Cook, 1985). Either negative exponential curves or straight lines were used to remove the effects of tree age (Fritts, 1976). If both of the curves were failed, a cubic smoothing spline with a 50% frequency-response cut-off of 80 years was applied. In addition, a univariate autoregressive model was used to remove the time series effect. The residual indices from autoregressive modeling of the detrended series were averaged by year with a robust mean calculation (Cook, 1985). The results were residual tree-ring chronologies, representing the common signal for the site. Descriptive statistics that were calculated for assessing the quality of the chronology included mean sensitivity (MS), standard deviation (SD), signal to noise ratio (SNR) and expressed population signal (EPS).

### Dendroclimatic analysis

In this study, correlation coefficients between tree-ring chronologies with monthly mean temperature and monthly precipitation derived from the MTCLM program were used to identify the relationships between radial growth variability of *A. faxoniana* and climate variables (Tardif et al., 2001). Because climatic conditions in previous growing season often influence the radial growth in the following year (Fritts, 1976), temperature and precipitation beginning in previous June until current September (from 1951 to 2009) were used to analyze the relationships between annual radial growth and climate variables. The analyses were produced using the DENDROCLIM 2002 software (Biondi and Waikul, 2004).

### Age structure and recruitment analysis

**Figure 3:**
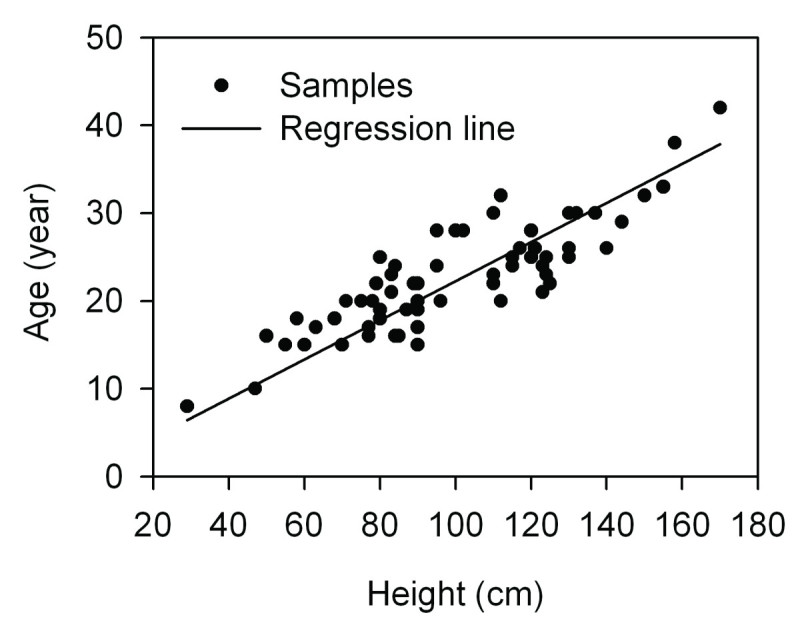
**Relationship between ages and height of** ***A. faxoniana*** **seedlings and saplings at the treeline ecotone in the Wanglang Nature Reserve.**

For the individual fir trees, the ages were determined using the cross-dated years from cores and the time to reach the coring height. If the cores passed close to the pith or missed inner parts, the number of rings missing from the pith was estimated by the geometric method (Duncan, 1989). Based on the relationship between age (*y*, year) and height (*x*, cm) of the fir seedlings/saplings (Figure 3):

1 $$y=0.180x+4.615\left(R^{2}=0.728,P<0.001,n=68\right)$$

the fir trees required 29 years to reach breast height (1.3 m above the ground). The ages of the seedlings and saplings were determined by the number of whorls and scars on the main stem (Dang et al*.*,^2009^). The ageing methodology may underestimate age by several years (Lv and Zhang, 2011). From the statistics of differences between the discs’ ages and the number of whorls/scars of the randomly uprooted seedlings and saplings, the results showed that only 20% of the samples were accurate. Of the samples, 15% were being underestimated one year, 20% were being underestimated two years and 45% were being underestimated three or more years. So the age frequency data for seedlings and saplings were smoothed using the following function (a method similar to the one of Daniels and Veblen, 2004):

2 $$x_{t}=0.20\left(f_{t}\right)+0.15\left(f_{t+1}\right)+0.20\left(f_{t+2}\right)+0.45\left(f_{t+3}\right)$$

where *x* is the smoothed age frequency, *f* is the original age frequency, and *t* is the year of seedling recruitment.

Rates of recruitment vs mortality cannot be differentiated from a static age structure alone. So we detrended the time series of annually recruited seedlings and saplings to account for mortality by the best-fit theoretical distributions, the exponential and power functions (Hett and Loucks, 1976). The difference between the theoretical age frequency and observed age frequency provided a time series of residuals (Szeicz and Macdonald, 1995). Then the “seedling residuals” were used to assess climate-recruitment relationships. The influence of climate change on recruitment was tested using Pearson’s correlation coefficients between recruitment and climate data derived from MTCLIM for the 1951–2009 periods (Daniels and Veblen, 2004; Jump et al., 2007). The climate parameters were the same as those described for the dendroclimatic analysis of the fir trees. The time period of current year January-December was used in this analysis (Jump et al., 2007).

## Results

### Chronologies and descriptive statistics

**Figure 4:**
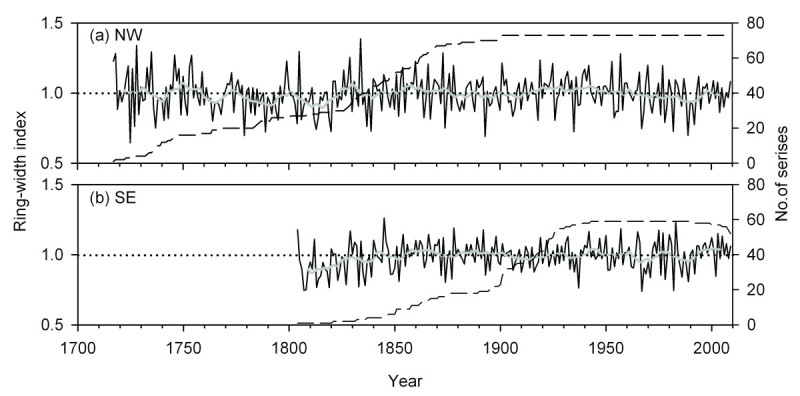
**Radial growth patterns of** ***A. faxoniana*** **at the treeline ecotones on the northwestern (a) and southeastern (b) aspects in the Wanglang Nature Reserve.** Thin line and thick line represent the annual series and 10-year smoothing spline of each ring-width chronology, respectively. Dash line represents the number of cores included in each residual chronology.

Following standard procedures, residual chronologies for *A. faxoniana* in the treelines on both the northwestern and southeastern aspects and the statistics are presented in Figure 4 and Table 1. Both chronologies for the two aspects showed similar patterns with characteristic narrow rings in the same pointer years such as 1893, 1935–1937, 1967, 1982 and 1989 (Figure 4). All the descriptive statistics are higher in the chronology for the northwestern aspect than in that for the southeastern aspect (Table 1). Although the MS and SD are not very high, the common interval analysis indicates high SNR and EPS for the two chronologies, respectively (Table 1).

**Table 1:** **Descriptive parameters of residual chronologies of** ***A. faxoniana*** **at the treeline ecotones on the northwestern (NW) and southeastern (SE) aspects of the Min Mountains in the Wanglang National Nature Reserve**

Plot code	NW	SE
**Chronology length**	**1715-2009**	**1803-2009**
Number of cores/trees	73/38	59/41
Mean ring width (mm)	0.991	1.001
Mean sensitivity (MS)	0.139	0.110
Standard deviation (SD)	0.124	0.096
Autocorrelation order 1 (AC1)	0.004	0.002
**Common interval time span**	**1873–2009**	**1926-2009**
Number of cores/trees	67/37	47/33
Mean interseries correlation	0.562	0.556
Signal-to-noise ratio (SNR)	18.658	13.719
Express population signal (EPS)	0.949	0.932

### Radial growth trends with climate

**Figure 5:**
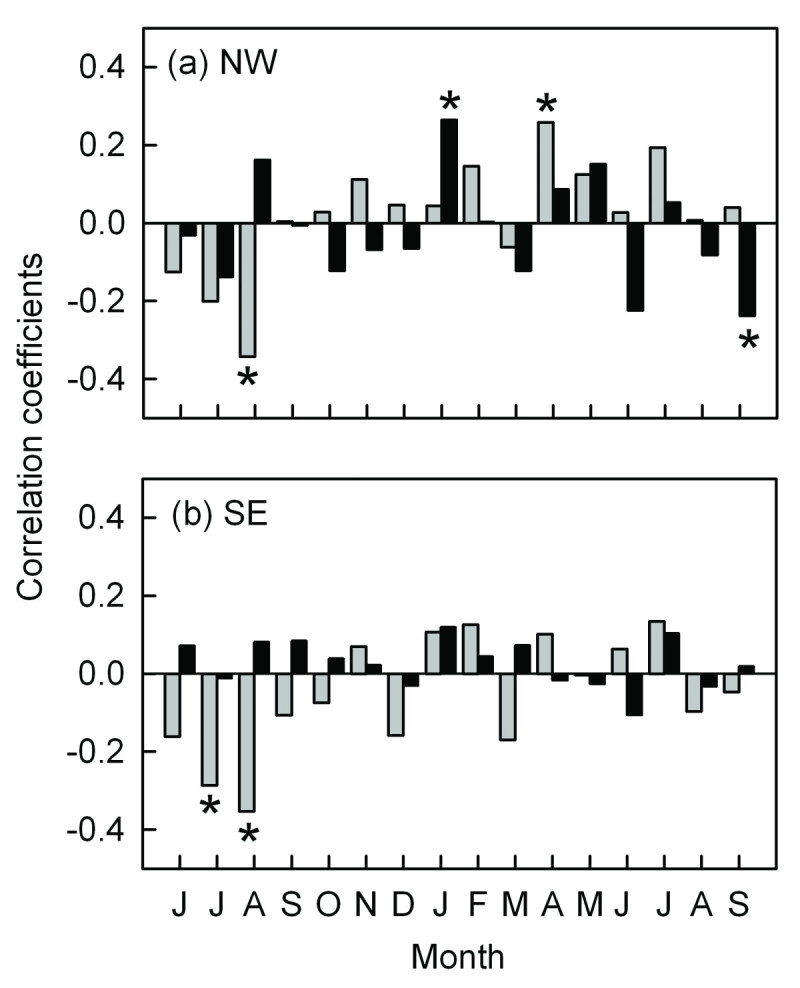
**Correlations between climate variables from 1951 to 2009 and chronologies for** ***A. faxoniana*** **at the treelines on the northwestern (a) and southeastern (b) aspects in the Wanglang Nature Reserve from June of the previous year to September of the current year.** The gray bars represent monthly mean temperature and the black bars represent monthly total precipitation. * *P* < 0.05.

The main climatic influence on the radial growth of *A. faxoniana* appears to be temperatures in the previous summer, especially in previous August, which were significantly negatively correlated with radial growth on both aspects (Figure 5). On the northwestern aspect, the radial growth of *A. faxoniana* was significantly negatively correlated with monthly mean temperature for the previous August, and was significantly positively correlated with monthly mean temperature for the current April (Figure 5a); and precipitation in the current January and September were significantly correlated positively and negatively with the current radial growth, respectively (Figure 5a). On the southeastern aspect, temperatures in the previous July and August were significantly negatively correlated with the radial growth (Figure 5b); and precipitation showed no significant correlation with ring-width indices (Figure 5b).

### Distribution of age structure

**Figure 6:**
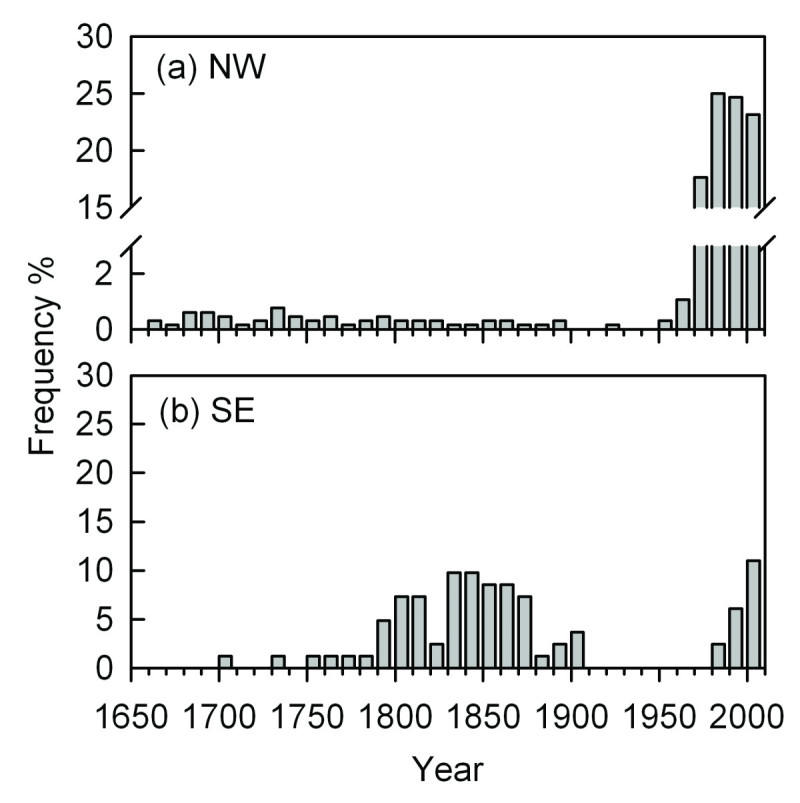
**Age frequency distribution patterns (10-year intervals) of** ***A. faxoniana*** **at the treelines on the northwestern (a) and southeastern (b) aspects in the Wanglang Nature Reserve.**

The age structures of *A. faxoniana* (10-year intervals) on both northwestern and southeastern aspects are shown in Figure 6. On the northwestern aspect, the 21-30-year-old age class (fir trees that established in the 1980s) accounted for the largest age class (25%). The majority of fir trees (90%) were found during the period from the 1970s to the 2000s and their ages ranged from 1 to 40 years (Figure 6a).

On the southeastern aspect, the 1-10-year-old age class (fir trees that established in the 2000s) formed the largest age class (11%). The majority of fir trees (78%) were successfully recruited into the treeline ecotone from 1750 s to 1900s (Figure 6b). However, there was no successful recruitment in successive decades from 1910s to 1970s.

### Recent recruitment and climate

The short-time recruitment records in recent decades did not match with the available meteorological records (1951–2009) in the treeline on the southeastern aspect (Figure 6b), so we only assessed the climate-recruitment relationships on the northwestern aspect.

**Figure 7:**
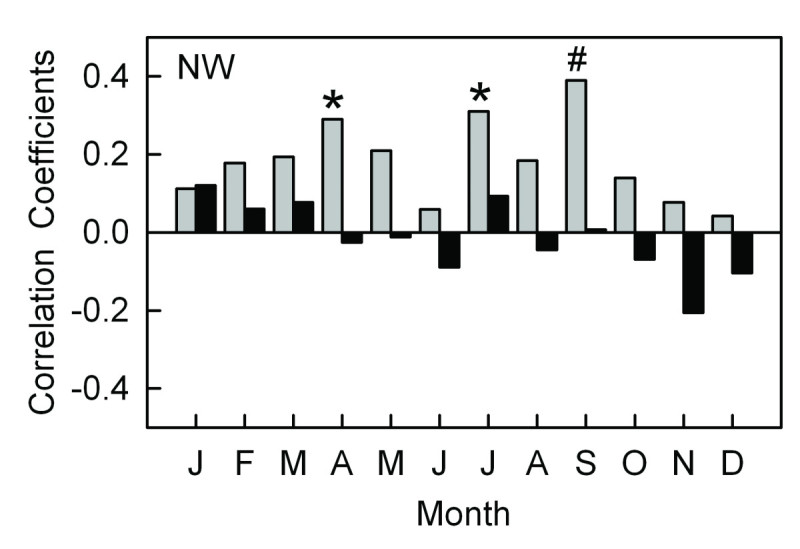
**Correlations between climate variables and recruitment residuals for** ***A. faxoniana*** **on the northwestern aspect from January to December (1951–2009).** Legends as for Figure 5. ^#^*P* < 0.01 and * *P* < 0.05.

During the last 60 years, significantly positive correlations existed between recruitment and monthly mean temperatures for the current April, July and September. However, monthly precipitation had no significant correlations with the recruitment (Figure 7).

## Discussion

### Correlations between radial growth and climate

Generally, the responses of radial growth to temperatures in previous summer were similar at the treeline ecotones on both the aspects. Significant negative correlations with previous July/August mean temperatures likely reflected the negative carry-over effects (Figure 5). Negative correlations between tree growth and previous-year July temperatures have been reported at high-elevation forests on southern (Liang et al., 2010) and southeastern (Lv and Zhang, 2011) QTP. High temperatures in the previous growing season (July/August) may reduce storage of assimilates for growth of next year (Liang et al., 2010). Moreover, fir radial growth was significantly positively correlated with monthly mean temperature in current April on the northwestern aspect (Figure 5a). Li et al. (2010) have also shown that radial growth of *A. faxoniana* was positively correlated with the temperature in current March at the treeline site in Wolong National Natural Reserve (30°53′N, 102°59′E), which lies west of our study area. Warmer temperatures early in the growing season benefit *A. faxoniana* growth by inducing early snowmelt and increasing available soil water (Wang et al., 2006; Dang et al., 2009), which causes early initiation of cambial activity and increases photosynthates (Splechtna et al., 2000; Case and Peterson, 2005).

The correlations between the precipitation and radial growth were different on both aspects (Figure 5). There was no significant correlation between precipitation and radial growth on the southeastern aspect (Figure 5b). On the northwestern aspect, the precipitation of the current January facilitated the radial growth (Figure 5a). This might represent relationships with snowpack and the subsequent effects on soil moisture (D’Arrigo et al. 2001. Significant negative correlation was found between radial growth and precipitation in the current September on the northwestern aspect, indicating that excessively sufficient precipitation will restrict the radial growth. Frequent rainfall in the end of the growing season of *A. faxoniana* might reduce solar radiation and effective photosynthesis, thereby, shorten the growing season (Wang et al., 2007; Jiang et al., 2010). The relationship between radial growth and precipitation on both the aspects might indicate that the precipitation was not the main limiting factor affecting radial growth during the main growing season.

The differential responses of radial growth to climate factors on both sites might be mainly due to the difference in micro-environmental conditions between the contrasting slope aspects. The studied area is dominated by the southeast monsoon (Pu et al., 2008; Yao et al., 2010). The southeastern aspect is wetter and warmer than the northwestern aspect, and also with stronger solar radiation. The sensitivity and response of treelines to climate variability may vary both in local and regional topographical conditions for the environmental variation (Holtmeier and Broll, 2005; Elliott and Kipfmueller, 2011). In our study, on the northwestern aspect with relatively weak solar radiation, the radial growth showed significantly negative correlation with precipitation in the end of the growing season (Figure 5a). The precipitation in January only favored the radial growth significantly on the northwestern aspect (Figure 5a) might because the difference of soil moisture on both the aspects. January snow might improve the soil moisture on the relatively drought northwestern slope in spring, therefore favored fir growth at the beginning of growing season.

### Recruitment and climate correlations on the northwestern aspect

The age structure of a stand can provide a fairly accurate picture of temporal variations in the recruitment rate (Kullman, 1991) with the dynamics of climate change (Payette and Filion, 1985), because tree recruitment is more sensitive than tree mortality to climate variability (Camarero and Gutiérrez, 2004). Most of the fir trees in the treeline on the northwestern aspect were younger than 40 years, but only about 10% were successfully recruited into the stand before 1960 (Figure 6a), which showed that fir recruitment had a sporadic mode from 1670 to 1960. In the Min Mountains, the fir trees possessed good soil seed banks at treeline ecotones (Fang, 2006), which suggests that seed production has not been an important influencing factor on the age-structure distribution (Wang et al., 2006; Dang et al., 2009). Cheng et al. (2005) have revealed that the high mortality of *A. faxoniana* at the treeline of the Min Mountains on the eastern edge of QTP might be controlled mainly by temperature, wind, snowpack depth and winter drought.

Recruitment was mainly affected by the temperatures in the spring, summer and early autumn seasons, and it was found to have significantly positive correlations with mean temperatures in April, July and September (Figure 7). Temperatures in spring facilitating fir seedling establishment were also found in the treeline ecotones of the Shennongjia Mountains (Dang et al., 2009). High temperatures in spring are very important for germination (Camarero and Gutiérrez, 1999) and might simulate tree recruitment from soil seed banks (Dang et al., 2009). The significant positive correlations between summer temperatures and recruitment were also reported from conifer seedlings in alpine treeline ecotone of the Snowy Range in Wyoming USA (Germino and Smith, 1999) and from *Abies spectabilis* forest in the alpine timberline of the Mt. Everest in southern QTP, China (Lv and Zhang, 2011). Higher summer temperatures will strengthen the photosynthesis rate, which could encourage both growth and nonstructural carbon storage for fir seedlings to survive harsh winter climate (Camarero and Gutiérrez, 1999).

### Recruitment on the southeastern aspect

There was a large gap in recruitment during the middle of the 20th century on the southeastern aspect (Figure 6). Competition from the dense bamboos might be mainly responsible for the rare recruitment (Dang et al., 2009). The bamboo cover in the southeastern plot (42.25%) was much higher than that in the northwestern plot (19.53%) (Zhao et al., 2012). Some studies have demonstrated that bamboos with a relatively high cover seem sufficient to impede tree establishment in subalpine forests (Takahashi, 1997; Holz and Veblen, 2006). In our study, the microenvironment on the southeastern aspect might be more suitable for bamboos’ cloning growth. The restriction of dense bamboos might exceed the facilitation of warm climate to the recruitment in recent decades.

### Radial growth versus recruitment

Over the most recent decades, the radial growth of *A. faxoniana* had no obvious increasing trends with small fluctuations on both the aspects (Figure 4). However, the recruitment has increased sharply, especially on the northwestern aspect (Figure 6a). Obvious increases of tree recruitment with the recent climate change were also found in other treeline ecotones, such as European Alps (Leonelli et al., 2011), Swedish Scandes (Kullman, 2005) and U.S. Rocky Mountain (Elliott and Kipfmueller, 2011). Our result was accordant with the treeline dynamics about *A. spectabilis* on the southern QTP (Lv and Zhang, 2011), but was different from those obtained in similar studies carried out in the central Tianshan Mountains (Wang et al., 2006) and the Shennongjia Mountains (Dang et al., 2009). Wang et al. (2006) and Dang et al. (2009) have found more radial growth but less recruitment with *Picea schrenkiana* and *Abies fargesii* in response to warm climate over several of the most recent decades. The different results suggest that the response of treeline ecotones to climate change varies with both local site conditions and the individual species (Luckman and Kavanagh, 1998).

At many upper treeline ecotones the climatic conditions that facilitate seedling recruitment are frequently similar to those conducive to radial growth of trees (Szeicz and Macdonald, 1995; Camarero and Gutiérrez, 1999; Jump et al., 2007; Dang et al., 2009). In our study the climatic conditions that facilitated the *A. faxoniana* seedling recruitment were also similar to those that enhanced the radial growth of fir trees. For example, high temperatures in current April enhanced the recruitment of *A. faxoniana* and facilitated the radial growth (Figures 5 and 7); more January precipitation in current year strengthened both seedling recruitment and radial growth (Figures 5 and 7).

## Conclusions

In summary, we investigated the climatic response of radial growth and recruitment of *A. faxoniana* in the treeline ecotones on the eastern edge of QTP. For the geographical novelty, QTP is attracting more and more attention on conducting similar studies (Liang et al., 2010; Lv and Zhang, 2011). In the future, continuous studies should be conducted, such as carrying out a second census within the same plots or setting up plots covering larger elevational gradients, in order to understand the dynamics of treeline position with the climatic variability on the eastern edge of QTP. Moreover, such studies will undoubtedly advance our knowledge of the other treelines on the eastern edge of QTP and reveal common and species-specific responses to climate change that may be exploited in conservation and protection efforts.

## Authors’ original submitted files for images

Below are the links to the authors’ original submitted files for images. Authors’ original file for figure 1 Authors’ original file for figure 2 Authors’ original file for figure 3 Authors’ original file for figure 4 Authors’ original file for figure 5 Authors’ original file for figure 6 Authors’ original file for figure 7
