# Supplementary figures and images for: Treeline dynamics in response to climate change in the Min Mountains, southwestern China
Source: Bot Stud. 2013 Aug 22;54:15. doi: 10.1186/1999-3110-54-15 (PMC5430343; doi:10.1186/1999-3110-54-15)

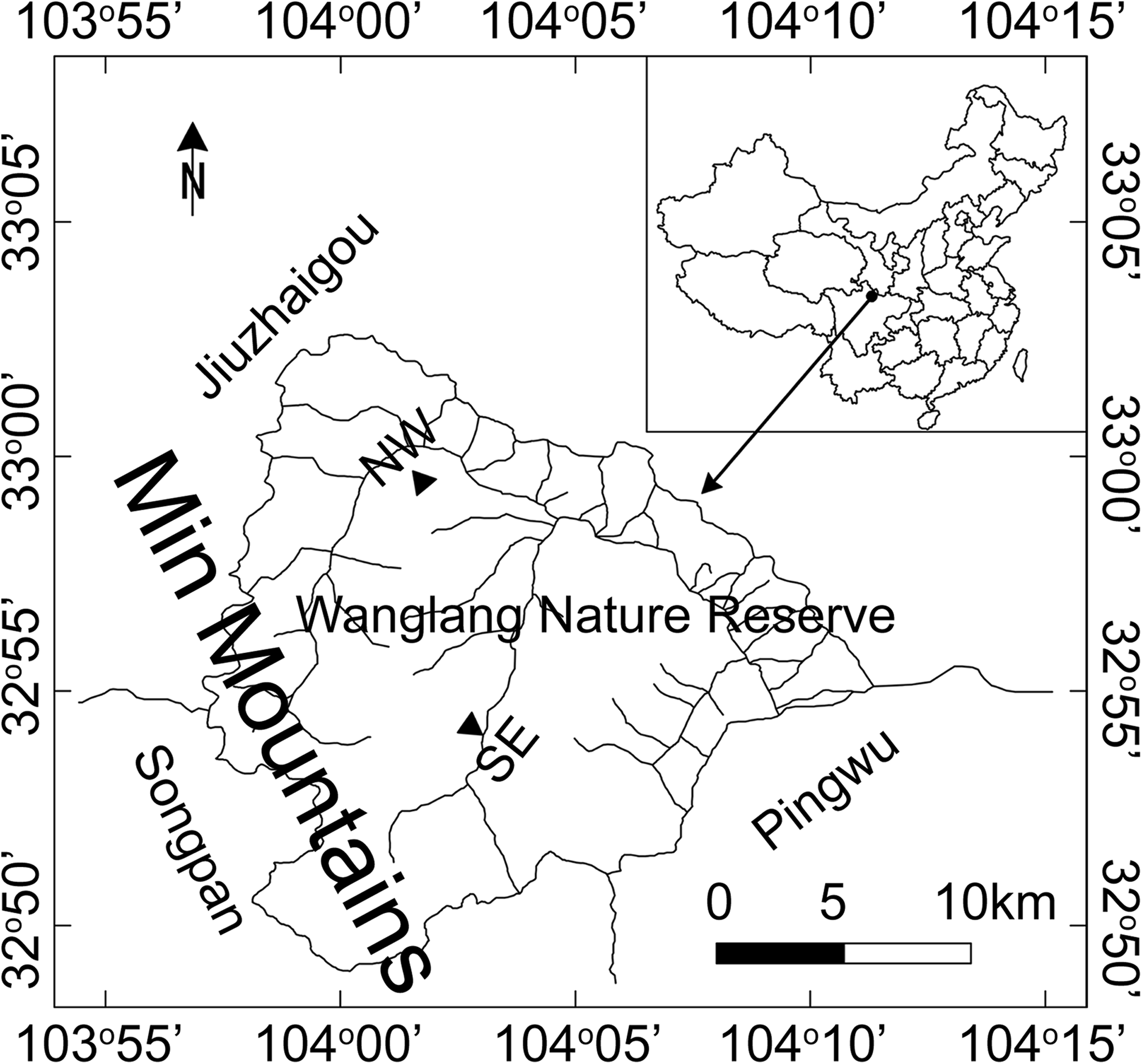

Supplement: Supplementary file 1 — Authors’ original file for figure 1 [file 40529_2011_13_MOESM1_ESM.tif]

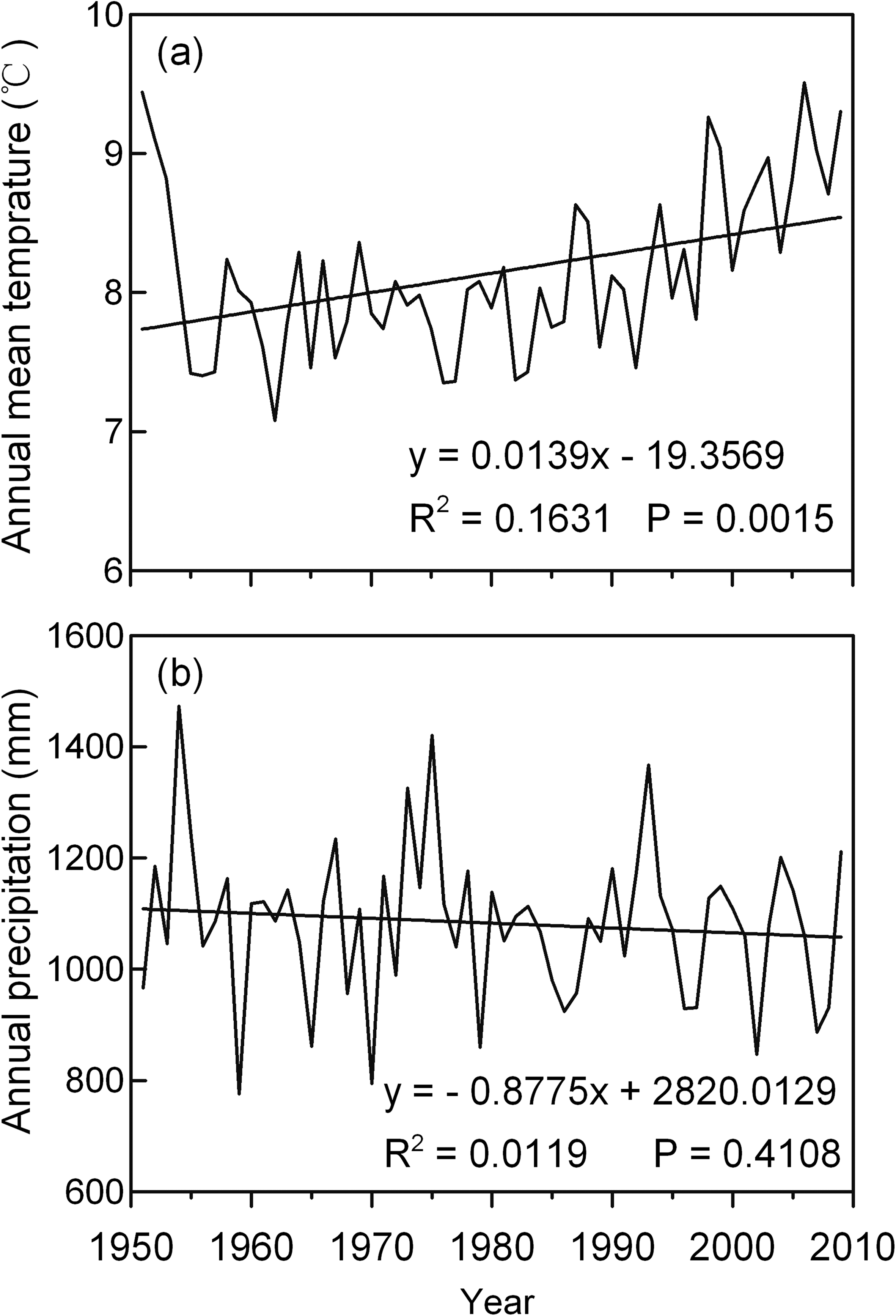

Supplement: Supplementary file 2 — Authors’ original file for figure 2 [file 40529_2011_13_MOESM2_ESM.tif]

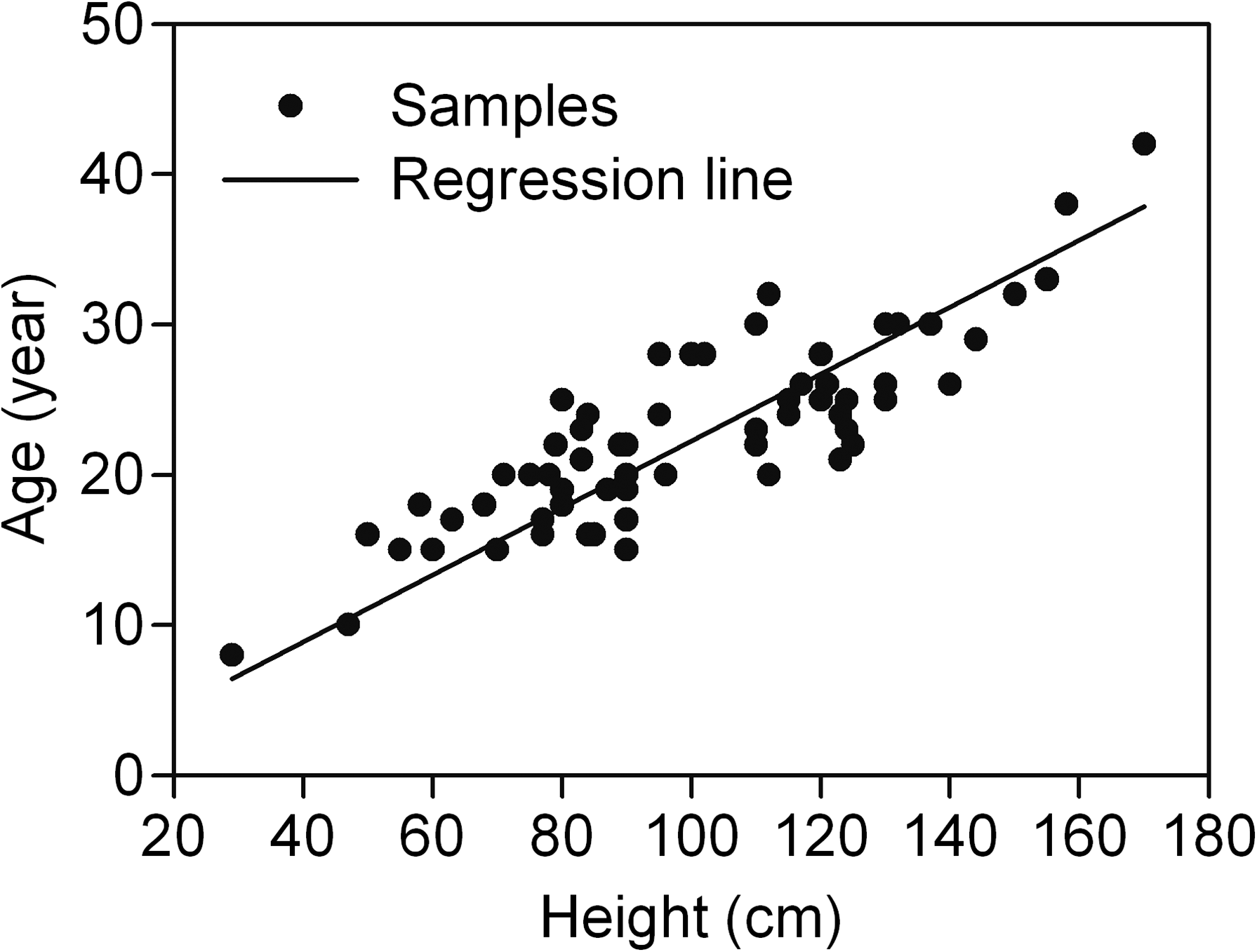

Supplement: Supplementary file 3 — Authors’ original file for figure 3 [file 40529_2011_13_MOESM3_ESM.tif]

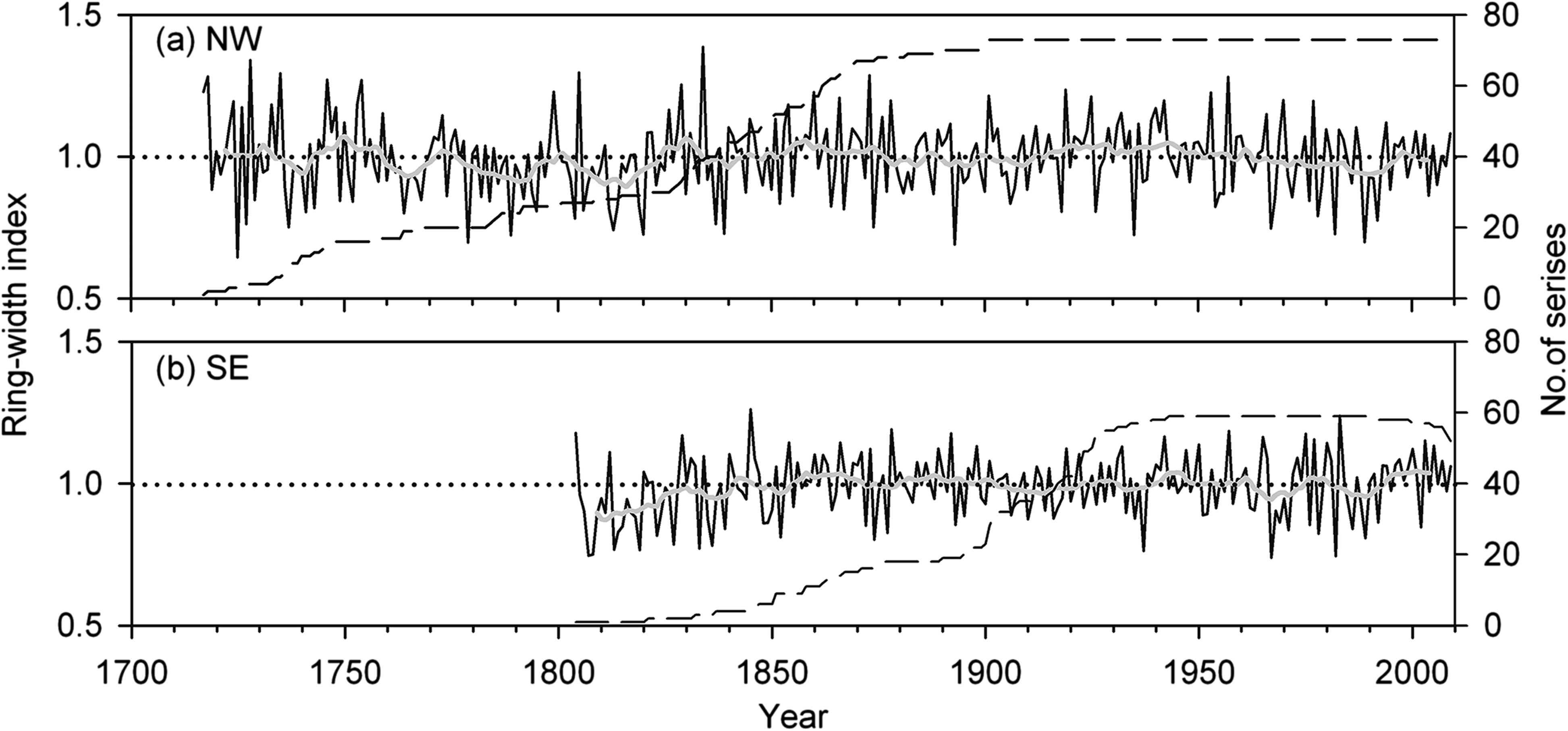

Supplement: Supplementary file 4 — Authors’ original file for figure 4 [file 40529_2011_13_MOESM4_ESM.tif]

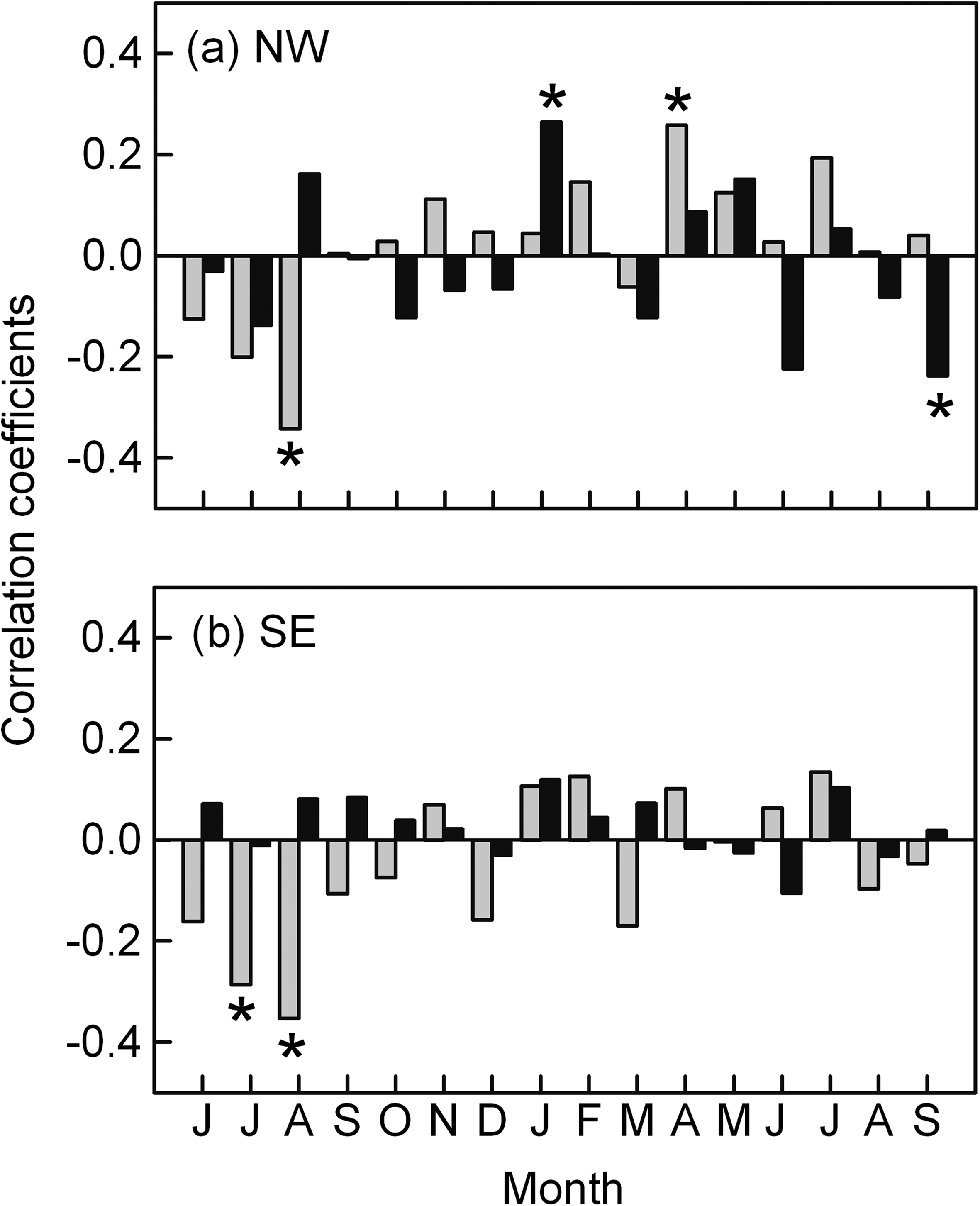

Supplement: Supplementary file 5 — Authors’ original file for figure 5 [file 40529_2011_13_MOESM5_ESM.tif]

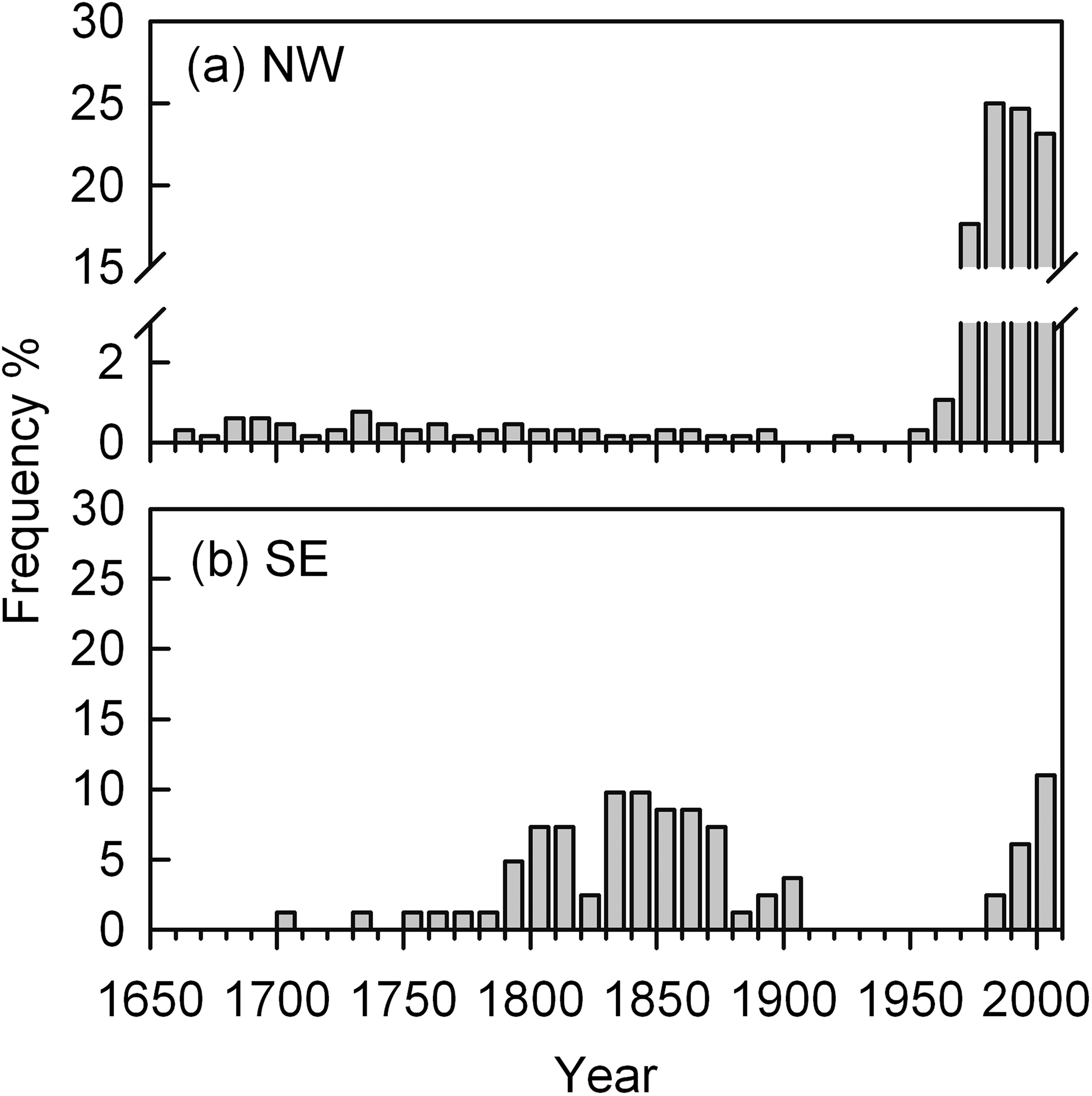

Supplement: Supplementary file 6 — Authors’ original file for figure 6 [file 40529_2011_13_MOESM6_ESM.tif]

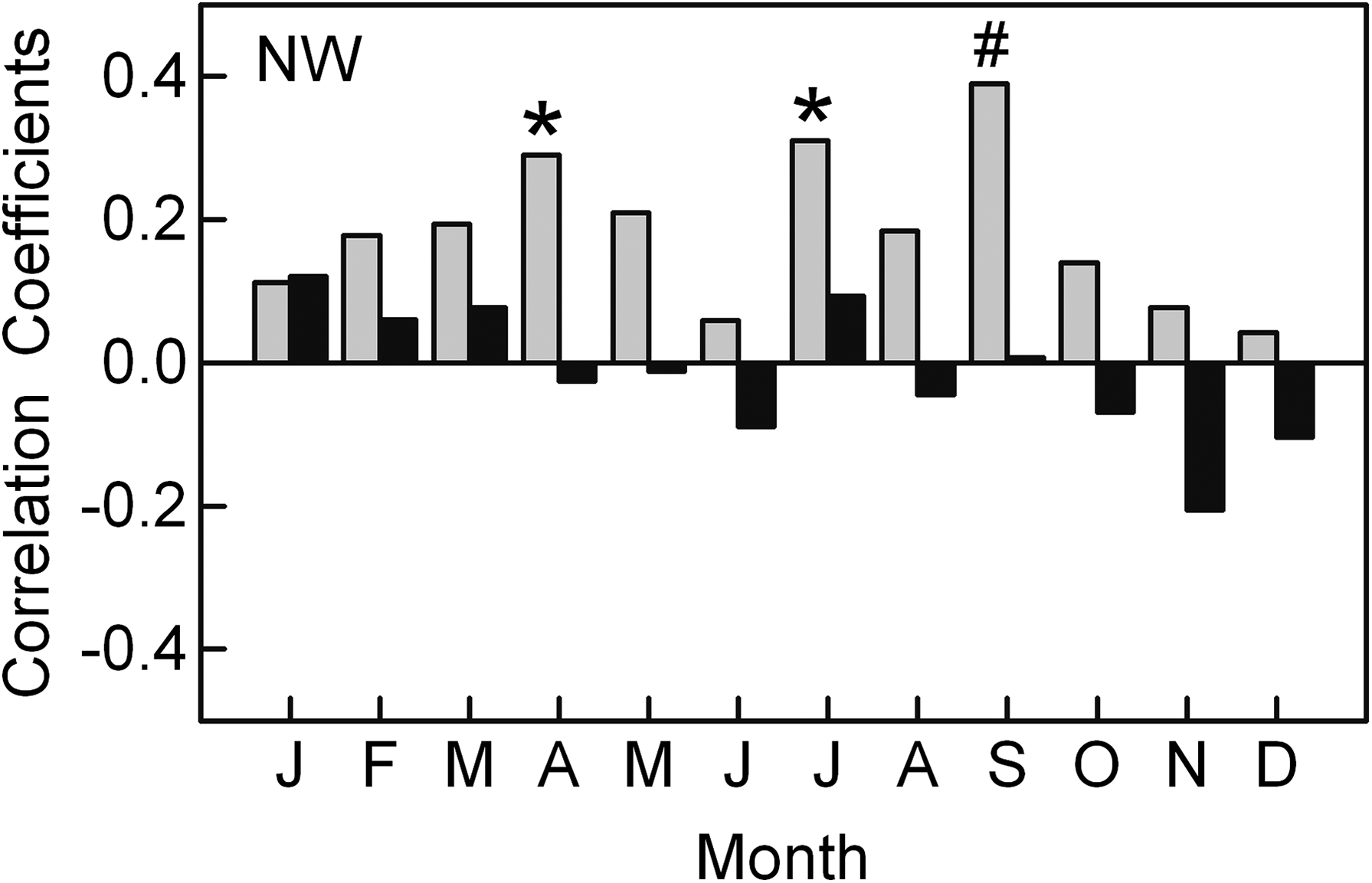

Supplement: Supplementary file 7 — Authors’ original file for figure 7 [file 40529_2011_13_MOESM7_ESM.tif]
